# Supplementary material for: A Novel Therapeutic Tumor Vaccine Targeting MUC1 in Combination with PD-L1 Elicits Specific Anti-Tumor Immunity in Mice
Source: Vaccines (Basel). 2022 Jul 8;10(7):1092. doi: 10.3390/vaccines10071092 (PMC9325010; doi:10.3390/vaccines10071092)

Figure S1: Transcriptional levels of MUC1 and PDL1 in LLC and Panc02 were detected by qPCR. LLC and Panc02 cells were infected with lentiviruses containing the gene sequences of human MUC1 and human PD-L1, and integrated into the genome of mouse tumor cells. The cells that were successfully transfected and stably expressed MUC1 and PDL1 were screened by puromycin resistance gene. After the transfection and drug screening, RNA was extracted from the cells and the transcription mRNA levels of MUC1 and PDL1 were detected by qPCR. ***P<0.001， ****P<0.0001

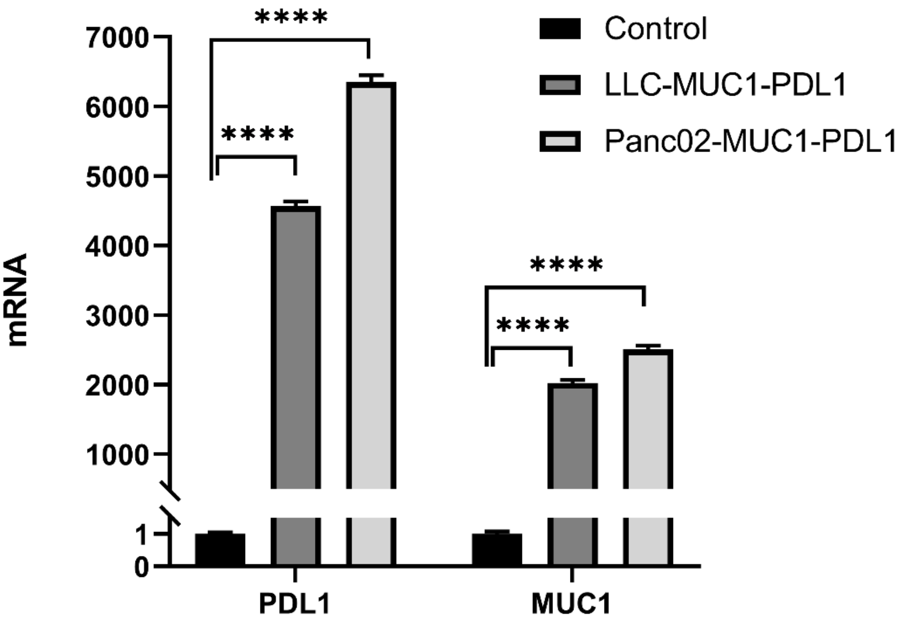

Supplement: Supplementary file 1 [file vaccines-10-01092-s001.zip › vaccines-1755384-supplementary.pdf]
